# Intrinsic calcium resonance and its modulation: insights from computational modeling

**DOI:** 10.3389/fncom.2025.1669841

**Published:** 2025-09-18

**Authors:** Rahul Kumar Rathour, Hanoch Kaphzan

**Affiliations:** Sagol Department of Neurobiology, Faculty of Natural Sciences, University of Haifa, Haifa, Israel

**Keywords:** computational model, hippocampal neurons, calcium resonance, voltage resonance, intrinsic calcium response dynamics

## Abstract

Hippocampal neurons generate membrane potential resonance due to specific voltage-gated ion channels, known as resonating conductances, which play crucial physiological roles. However, it is not known whether this phenomenon of resonance is limited to membrane voltage or whether it propagates through molecular signaling components such as calcium dynamics. To test this, we first utilized a single-compartment model neuron to study the oscillatory intrinsic calcium response dynamics of hippocampal model neurons, and the effects of T-type calcium channel kinetics on the voltage and calcium resonance. We found that in the presence of T-type calcium channels, our model neuron sustained a strong calcium resonance compared to voltage resonance. Unlike voltage resonance, calcium resonance frequency was largely independent of conductance magnitude, and the two types of resonance were dissociated, meaning independent of each other. In addition, we studied the effects of A-type K^+^-channels and h-channels in conjunction with T-type calcium channels on calcium resonance, and showed that these two types of channels differentially affect calcium resonance. Finally, using a multi-compartmental morphologically realistic neuron model, we studied calcium resonance along the somato-apical dendritic axis. Using this model, we found that calcium resonance frequency remains almost constant along the somato-apical trunk for the most part, and only toward its terminal end, the calcium resonance frequency was increased. Nonetheless, this increase was lesser compared to the increase in voltage resonance frequency. Our study opens new horizons in the field of molecular resonance, and deepen our understanding concerning the effects of frequency-based neurostimulation therapies, such as transcranial alternating current stimulation (tACS).

## Introduction

Hippocampal neurons are endowed with a plethora of voltage-gated ion channels (VGICs) (Johnston and Narayanan, 2008; Johnston et al., 1996; Lai and Jan, 2006; Migliore and Shepherd, 2002). These VGICs play a critical role in neuronal physiology (Branco and Hausser, 2011; Branco et al., 1979; Hutcheon and Yarom, 2000; Llinas, 1988; London and Hausser, 2005; Magee, 2000; Marder et al., 1996; O’Donnell and Nolan, 2011; Remme et al., 2010; Sjostrom et al., 2008; Spruston, 2008; Wang, 2010). Any aberration in the physiological functioning of these VGICs could lead to pathological conditions (Bernard et al., 2007) and impaired information encoding (Sjostrom et al., 2008; Zhang and Linden, 2003; Kim and Linden, 2007; Mozzachiodi and Byrne, 2010; Turrigiano and Nelson, 2000; Remy et al., 2010). One of the important aspects of certain VGICs is that they enable neurons to discriminate the inputs based on their frequency of arrival (Hutcheon and Yarom, 2000). When a neuron receives inputs of various frequencies it produces maximal voltage response at a distinct non-zero input frequency. This phenomenon is known as membrane potential resonance and VGICs governing this phenomenon are known as the resonating conductance. Presence of resonance serves important functions in neuronal physiology (Hutcheon and Yarom, 2000; Narayanan and Johnston, 2007; Narayanan and Johnston, 2008; Rathour et al., 2016). However, some aspects concerning resonance in neurons are yet unclear. For example, it is not fully understood whether this phenomenon of resonance is limited to voltage signals or does it also propagate via signaling cascades.

This is particularly important to understand given that neuronal physiology and plasticity are heavily coupled to the activation of signaling components. Even if the phenomenon of resonance propagates through the signaling components, it is not established whether it gets transferred to the next step of the signaling cascade or does every step possess some intrinsic mechanism to generate molecular resonance on its own. Molecular resonance denotes a frequency-selective response in an intracellular signaling variable, which in our case is cytosolic Ca^2+^ levels that will result in igniting downward calcium-dependent signaling. Hence, we aimed to examine the relations between input current frequency, voltage resonance and calcium resonance, which is coupled to signaling cascades, i.e., molecular resonance.

As a first step to answer these questions, we focused on calcium dynamics of the neuron, given that calcium is an important intracellular messenger and act as a link between voltage dynamics and signaling cascade (Clapham, 2007; Berridge, 1998; Augustine et al., 2003). On the one hand, calcium is an ion (Ca^2+^) that its conductance affects the membranal voltage dynamics, while on the other hand, calcium ions are critical intracellular signaling molecules that trigger the initiation of most neuronal signaling cascades. In neurons, transient elevations in cytosolic Ca^2+^ levels play a central role in regulating processes such as synaptic transmission, plasticity, gene expression, and neurotransmitter release (Augustine et al., 2003; Bootman et al., 2001; Rose and Konnerth, 2001). By acting as a second messenger, Ca^2+^ coordinates the activation of specific downstream effectors, enabling the integration of electrical and chemical signals essential for neuronal communication and adaptive responses in neural circuits.

In the herein study we focused on T-type calcium channels as the source of calcium in our model neuron. Our choice to focus on T-type calcium channels was governed by the fact that these VGICs are an important source of calcium dynamics in pyramidal neurons, they are present in high density in dendrites, they induce voltage resonance, and are considered the most important calcium channels for induction of voltage resonance in neurons (Hutcheon and Yarom, 2000; Clapham, 2007; Berridge, 1998; Bootman et al., 2001; Magee and Johnston, 1995). In addition, the T-type calcium channels are low threshold calcium channels, which enables them to affect calcium concentrations even with weak subthreshold membrane polarization, making them significant contributors for calcium dynamics both in physiological oscillations and neurostimulation therapeutic strategies.

To this end, we define calcium resonance frequency as follows: when a neuron receives inputs at different frequencies then cytosolic calcium level reaches maximum at a particular input frequency owing to calcium entry from voltage gated calcium channels. This frequency is known as calcium resonance frequency and the phenomenon is known as calcium resonance.

Our study shows that T-type calcium channels are sufficient to induce calcium resonance on their own, and that calcium resonance frequency was largely independent of T-type calcium conductance magnitude but is modulated by the presence of A-type K^+^ and h channels. Moreover, we show that intrinsic calcium response dynamics did not simply follow the voltage dynamics rather there is a dissociation between the two.

## Materials and methods

**Figure 1:**
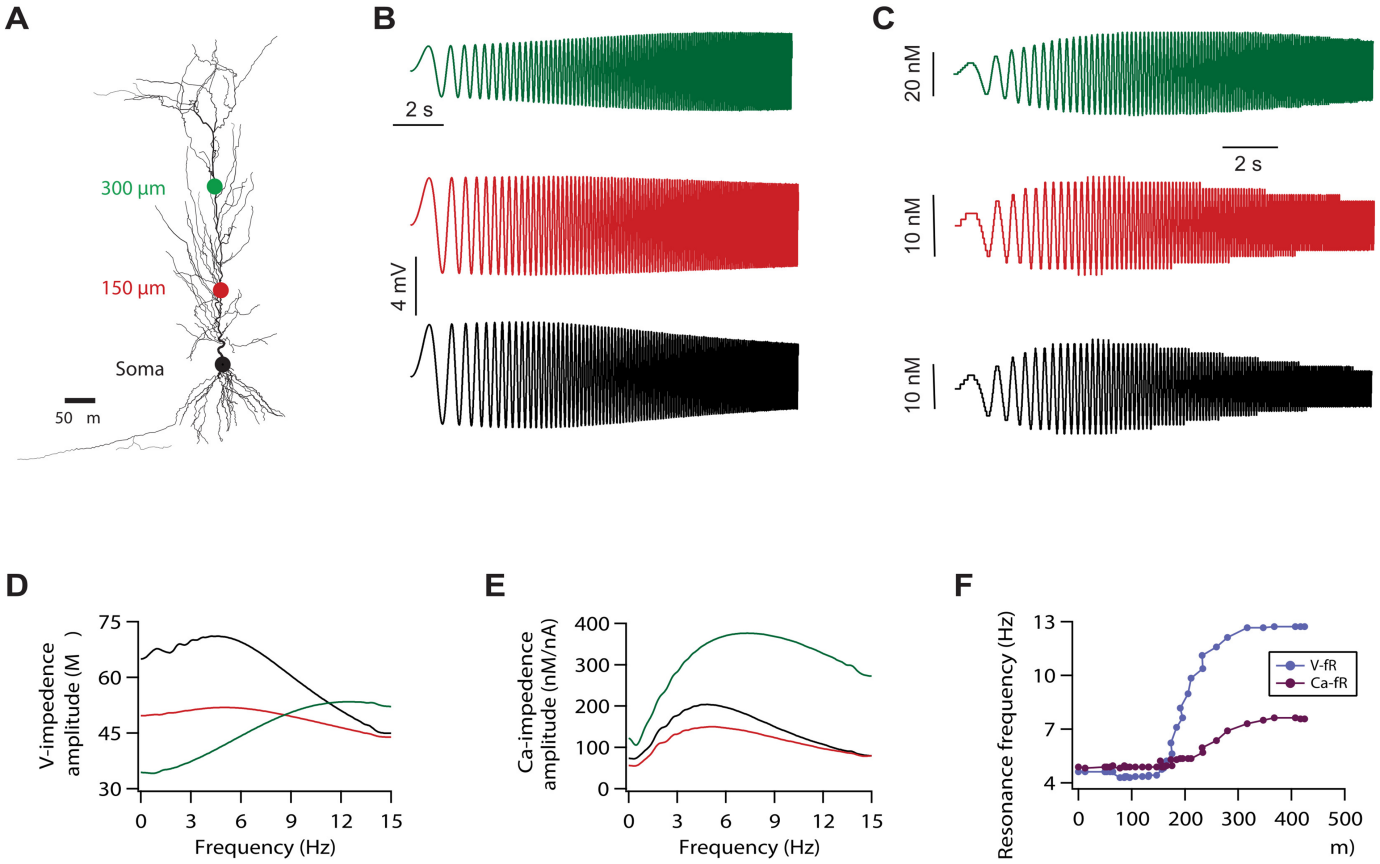
Calcium resonance frequency along the somato-apical trunk in a morphologically realistic model. **(A)** A morphologically realistic model of neuron used in this study. **(B)** Voltage traces, in response to a chirp stimulus, recorded from three different locations along the somato-apical dendritic trunk. Color matched recording locations are shown in **A**. **(C)** Calcium traces in response to the chirp stimulus recorded from three different locations along the somato-apical dendritic trunk. Color matched recording locations are shown in **A**. **(D)** Impedance amplitude profiles derived from the traces shown in **B**. **(E)** Calcium-related impedance amplitude profiles derived from the traces shown in **C**. **(F)** Calcium (magenta) and voltage (cyan) resonance frequency maps along the somato-apical dendritic trunk.

The study employed two distinct models. The first model was a single-compartment model (Supplementary Figure S1A) and was used to study basic mechanism of calcium resonance and its dependence on other ion channels. The second model was a multi-compartmental morphologically realistic model (Figure 1A), and was used to study calcium resonance properties along the somato-apical dendritic axis.

The single-compartment model was made of a single cylinder with the dimensions of length and diameter set at 60 μm each. Specific membrane resistance, *R*_m_, was set at 11 kΩ·cm^2^ while specific membrane capacitance, *C*_m_, was 1 μF/cm^2^. Axial resistance was 100 Ω·cm. This set of passive membrane parameters produced an input resistance of 97 MΩ.

A morphologically realistic, 3D reconstructed, rat hippocampal CA1 pyramidal neuron (*n123*), obtained from Neuromorpho.org (Ascoli et al., 2007) was used as the substrate for simulations. Morphology and modeling parameters of passive membrane properties and voltage-gated ion channels (VGICs) were the same as those used in previous studies (Rathour and Kaphzan, 2023; Rathour and Kaphzan, 2022; Rathour and Kaphzan, 2024), originally derived from (Rathour and Narayanan, 2014) and are detailed below.

### Passive membrane properties

Passive membrane parameters were set so the model neuron was able to capture experimental statistics of various measurements (Narayanan and Johnston, 2007; Narayanan and Johnston, 2008; Migliore et al., 1999; Johnston et al., 1999; Hoffman et al., 1997). Explicitly, specific membrane capacitance (*C*_m_) was set at 1 μF/cm^2^ across the entire morphology. Specific membrane resistivity (*R*_m_) and intracellular resistivity (*R*_a_) were distributed non-uniformly and varied along the somato-apical trunk as functions of the radial distance of the compartment from the soma (*x*) using the following formulation:


(1) $$R_{m}(x)=R_{m}-\max+\frac{\left(R_{m}-\min-R_{m}-\max\right)}{1+\exp((R_{m}-d-x)/R_{m}-k)}$$

(2) $$R_{a}(x)=R_{a}-\max+\frac{\left(R_{a}-\min-R_{a}-\max\right)}{1+\exp((R_{a}-d-x)/R_{a}-k)}$$

where *R_m_*-max = 125 k*Ω*/cm^2^ and *R_a_*-max = 120 Ω/cm were default values at the soma, and *R_m_*-min = 85 kΩ/cm^2^ and *R_a_*-min = 70 Ω/cm were values assigned to the terminal end of the apical trunk (which was ~425 μm distance from the soma for the reconstruction under consideration). The other default values were: *R_m_-d* = *R_a_-d* = 300 μm, *R_m_-k* = R*_a_-k* = 50 μm; *R_a_-k* = 14 μm. The basal dendrites and the axonal compartments had somatic *R_m_* and *R_a_*. Model neuron with these distributions of passive membrane properties was compartmentalized using d_λ_ rule (Carnevale and Hines, 2006) to ensure that each compartment was smaller than 0.1λ_100_, where λ_100_ was the space constant computed at 100 Hz. This produced a total of 809 compartments in the model neuron.

### Channel kinetics

The multi-compartmental model neuron expressed five conductance-based voltage-gated ion channels (VGICs): Na^+^, *A*-type K^+^ (K_A_), delayed rectifier K^+^ (K_DR_), *T*-type Ca^2+^ (Ca_T_), and hyperpolarization-activated cation non-specific *h* (HCN) channels. Na^+^, K_DR_, and K_A_ channels were modeled based upon previous kinetic schemes (Migliore et al., 1999), and *h* channels were modeled as in Poolos et al. (2002). *T*-type Ca^2+^ channels kinetics was taken from Shah et al. (2008). Na^+^, K^+^, and *h* channels models were based upon Hodgkin–Huxley formalism and had reversal potentials 55, −90, and −30 mV, respectively. The Ca_T_ current was modeled using the Goldman–Hodgkin–Katz (GHK) formulation with the default values of external and internal Ca^2+^ concentrations set at 2 mM and 100 nM, respectively. The Densities of Na^+^ and K_DR_ conductances were kept uniform across the neuronal arbor, whereas the densities of h, Ca_T_, and K_A_ channel conductances increased on the apical side with an increase in distance from the soma (Magee and Johnston, 1995; Hoffman et al., 1997; Magee, 1998). The basal dendritic compartments had somatic conductance values, with distribution of K_A_ channels as well. Calcium decay kinetics was modeled as in Narayanan and Johnston (2010).

K_A_ conductance was set as a linearly increasing gradient as a function of radial distance from the soma, *x* (Hoffman et al., 1997), using the following formulation:


(3) $${\bar{g}}_{\mathrm{KA}}(x)=A-g_{B}\;(1+A-\mathrm{Fx}/100)$$

where somatic ${\bar{g}}_{\mathrm{KA}}$ was 3.1 mS/cm^2^, and *A*-*F* (=8) quantified the slope of this linear gradient. In order to incorporate experimental observations related to differences in half-maximal activation voltage (*V*_1/2_) between the proximal and the distal K_A_ channels in CA1 pyramidal cells (Hoffman et al., 1997), two distinct models of K_A_ channels were adopted. A proximal model was used for compartments with radial distances less than 100 μm from the soma, and beyond that point a distal *A*-type K^+^ conductance model was used.

The increase in maximal *h* conductance along the somato-apical axis as a function of radial distance from the soma, *x*, was modeled using the following formulation:


(4) $${\bar{g}}_{h}(x)=h-g_{B}\;(1+\frac{h-F}{1+\exp((h-d-x)/h-k)})$$

where *h*-*g*_b_ denotes maximal *h* conductance at the soma, set to be 25 μS/cm^2^, and *h*-*F* (=12) formed fold increase along the somato-apical axis. Half-maximal distance of ${\bar{g}}_{h}$ increase, *h-d* was 320 μm, and the parameter quantifying the slope, *h-k* was 50 μm. To accommodate the experimental observations regarding changes in *V*_1/2_ of the activation of *h* conductance at various locations along the somato-apical trunk (Magee, 1998), the half-maximal activation voltage for *h* channels was −82 mV for *x* ≤ 100 μm, linearly varied from −82 mV to −90 mV for 100 μm ≤ *x* ≤ 300 μm, and −90 mV for *x* > 300 μm.

Although direct experimental evidence for CaT distribution along the CA1 apical dendrite is lacking, studies suggest a distal increase in expression/conductance. While other models are possible, we chose a sigmoidal profile for biological plausibility, capturing the gradual rise and distal saturation previously modeled (Rathour and Narayanan, 2014). Thus, Ca_T_ conductance was modeled as a sigmoidal function of the radial distance from the soma, *x*:


(5) $${\bar{g}}_{\mathrm{CaT}}(x)=T-g_{B}\;(1+\frac{T-F}{1+\exp((T-d-x)/T-k)})$$

where *T*-*g*_B_ denotes maximal Ca_T_ conductance at the soma, set to be 80 μS/cm^2^, and *T*-*F* (=30) formed fold increase along the somato-apical axis. Half-maximal distance of ${\bar{g}}_{\mathrm{CaT}}$ increase, *T-d* was 350 μm, and the parameter quantifying the slope, *T-k* was 50 μm. These parametric constraints accounted for the experimental constraints on the coexistence of the six functional maps along the same somato-apical trunk (Narayanan and Johnston, 2012).

**Figure 2:**
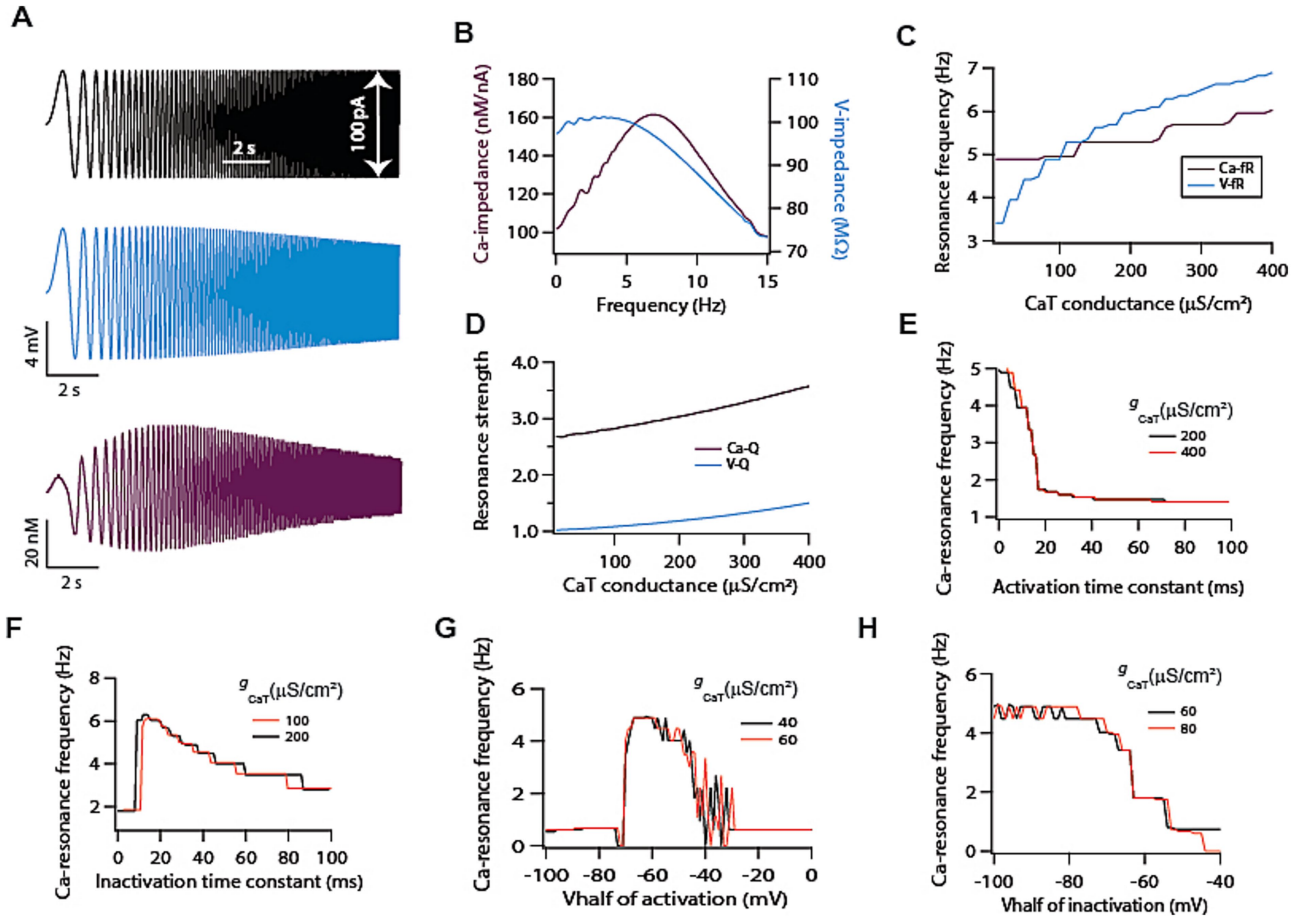
T-type calcium channels induce intrinsic calcium resonance in model neurons. **(A)** Representative traces of chirp stimulus (black) and corresponding voltage (cyan) and calcium (magenta) response. **(B)** Voltage (cyan) and calcium (magenta) related impedance amplitude profiles, derived from traces shown in **A**. **(C)** Dependence of voltage and calcium resonance frequencies on T-type calcium conductance. **(D)** Dependence of voltage and calcium resonance strengths on T-type calcium conductance. **(E)** Increasing activation time constant of T-type calcium conductance causes a decrease in calcium resonance frequency. **(F)** Increasing inactivation time constant of T-type calcium conductance causes a decrease in calcium resonance frequency. **(G)** Shifting *V*_1/2_ of activation of T-type calcium conductance produces bell shaped like curve for calcium resonance frequency. **(H)** Shifting *V*_1/2_ of inactivation of T-type calcium conductance in depolarized direction reduces calcium resonance frequency.

Subthreshold voltage/calcium response dynamics of the model to oscillatory inputs was characterized by injecting a chirp stimulus: a sinusoidal current wave with constant amplitude (50 pA) with frequency linearly increasing from 0 to 15 Hz in 15 s (Figure 2A). The Fourier transform of the voltage/calcium response was divided by the Fourier transform of the chirp stimulus to obtain the complex valued voltage impedance *Z*_V_(*f*) or calcium impedance *Z*_Ca_(*f*), as a function of frequency *f*. The impedance amplitude profile of voltage/calcium was then estimated as the magnitude of this impedance (Figure 2B). The frequency at which |*Z*(*f*)| reached its maximum value was measured as the resonance frequency (*f*_R_). Resonance strength (*Q*) was measured as the ratio of the maximum impedance amplitude to the impedance amplitude at 0.5 Hz (Hu et al., 2002).

Impedance profiles were computed as follows:


(6) ∣Z(f)∣=(Re(Z(f)))2+(Im(Z(f)))2

All simulations were performed using NEURON simulation environment (v8.0). For all simulations, the temperature was set at 34°C, and ion-channels kinetics was appropriately adjusted based upon experimentally determined q10 factors. The integration time constant, for solving various differential equations, was set to be 25 μs. Membrane potential was fixed at −65 mV and simulations were run at this potential. Data analyses were done using custom-built software written within IGOR Pro (v8.0) (Wavematrics).

## Results

Subthreshold intrinsic calcium response dynamics was studied in hippocampal model neurons. First, using single compartmental model, role of T-type calcium channels was explored in shaping the calcium response dynamics. Thereafter, T-type calcium channels were co-expressed with either hyperpolarization activated cation non-specific h current (*I*_h_) or A-type K^+^ current to study the effect of these channels on T-type calcium current mediated calcium response dynamics. Finally, using a morphologically realistic model, expressing gradients of ion channels and constrained by various physiological measurements, we explored dendritic calcium response dynamics.

### T-type calcium channels cause intrinsic calcium resonance

It is well established that T-type calcium channels can sustain voltage resonance on its own and can induce intrinsic membrane potential oscillation in various cell types (Hutcheon and Yarom, 2000). However, it is not established whether such voltage resonance can induce calcium resonance. To test this, we employed a single compartmental model (Supplementary Figure S1A) expressing only T-type calcium channels. With the delivery of chirp stimulus (Figure 2A; black trace) in this model, we simultaneously recorded the voltage (Figure 2A; cyan trace) and calcium concentration levels (Figure 2A; magenta trace) signals. As expected, and as previously demonstrated (Rathour and Narayanan, 2012), the voltage signal exhibited weak resonance (V-Q = 1.02, Figure 2B; cyan trace). Contrary to this, when we analysed calcium signal, we found that it exhibited strong resonance (Ca-Q = 2.52, Figure 2B; magenta trace). A possible explanation for this lies in the kinetics of T-type calcium channels. At lower frequencies, these channels would activate but would have enough time to inactivate owing to the slow change in membrane potential thus limiting the calcium response. On the other hand, at high frequencies these channels do not have enough time to activate, which also reduces calcium response. Only at intermediate frequencies where activation and inactivation kinetics balance each other, the calcium response reaches maximum value, hence giving rise to calcium resonance.

So far, our analysis showed that calcium resonance was strong compared to voltage resonance and peak calcium resonance frequency was higher compared to voltage peak resonance frequency for the same level of T-type calcium conductance (Figure 2B). This suggests calcium signal does not simply follow voltage signal rather there is a dissociation between the two signals. In order to understand this dissociation, we computed both the resonance frequencies for the range of T-type calcium conductance (Figure 2C). As expected, voltage resonance frequency increased monotonically with increase in T-type calcium conductance. To our surprise, calcium resonance frequency showed only small increase and was largely independent of T-type calcium conductance magnitude (Figure 2C). This clearly shows some dissociation between T-type calcium channels mediated voltage and calcium dynamics, and that calcium response dynamics does not simply follow the voltage response dynamics. Next, we looked at the resonance strength. Our previous analysis showed that calcium response dynamics exhibited strong resonance compared to voltage response dynamics. However, we wanted to determine whether this difference extends to the entire range of conductance values. To test this, we computed resonance strengths of voltage and calcium signals for the range of conductance values. We found that for the entire tested range of conductance values calcium resonance strengths were higher compared to voltage resonance strengths (Figure 2D). Moreover, both the resonance strengths increased with the increase in conductance values (Figure 2D).

Next, we turned our attention toward voltage dependent parameters of T-type calcium channels for determining calcium resonance frequency. Given that these parameters show neuron to neuron variability, therefore, it is important to understand the dependence of calcium resonance on these parameters. When we analysed calcium resonance frequency with respect to channels activation/inactivation time constants we found that increasing either time constant led to reduction in calcium resonance frequency (Figures 2E,F). On the other hand, when we analysed dependence of calcium resonance on *V*_1/2_ of activation/inactivation of T-type calcium channels we found that *V*_1/2_ of activation of T-type calcium conductance produced bell shaped like curve for calcium resonance frequency (Figure 2G). While Shifting *V*_1/2_ of inactivation of T-type calcium channels in depolarized direction reduces calcium resonance frequency (Figure 2H). These dependencies of calcium resonance frequency on T-type calcium channels parameters were not similar to T-type calcium channel mediated voltage resonance frequency (Rathour and Narayanan, 2012).

### Dependence of calcium resonance on passive parameters

Apart from voltage dependent parameters, other parameters could potentially modify T-type calcium channels-mediated calcium dynamics. Prominent among these is the calcium decay kinetics. Calcium decay time constant define the rate of removal of calcium from the neuron and has been shown to exhibit neuron to neuron variability (Dana et al., 2019). Therefore, we tested the dependence of calcium resonance frequency on calcium decay time constant. We found that with increase in calcium decay time constant, calcium resonance frequency is reduced (Supplementary Figure S1B). Apart from calcium decay kinetics, passive parameters could also have prominent role in defining neuronal resonance (Rathour and Narayanan, 2012). Therefore, we examined the involvement of these parameters in defining calcium resonance. We found that increased membrane resistance induced a small increase in calcium resonance frequency (Supplementary Figure S1C). On the other hand, increasing the membrane capacitance led to a decrease in calcium resonance frequency (Supplementary Figure S1D).

### Role of h channels and A-type K^+^ channels in determining calcium resonance

So far, we focused on T-type calcium channels and its associated parameters along with other passive parameters in determining calcium resonance frequency. But neurons express various other types of voltage gated ion channels along with T-type calcium channels (Johnston and Narayanan, 2008; Johnston et al., 1996). Prominent among these are h channels and A-type K^+^ channels. These voltage-gated ion channels have overlapping voltage activation ranges and are co-expressed with T-type calcium channels in the dendrites of CA1 neurons (Narayanan and Johnston, 2012). Co-expression of voltage-gated ion channels could lead to competition/cooperation among them, collectively shaping neuronal physiology (Rathour and Narayanan, 2012). Therefore, it is important to understand the role of these voltage-gated ion channels in determining calcium resonance frequency.

**Figure 3:**
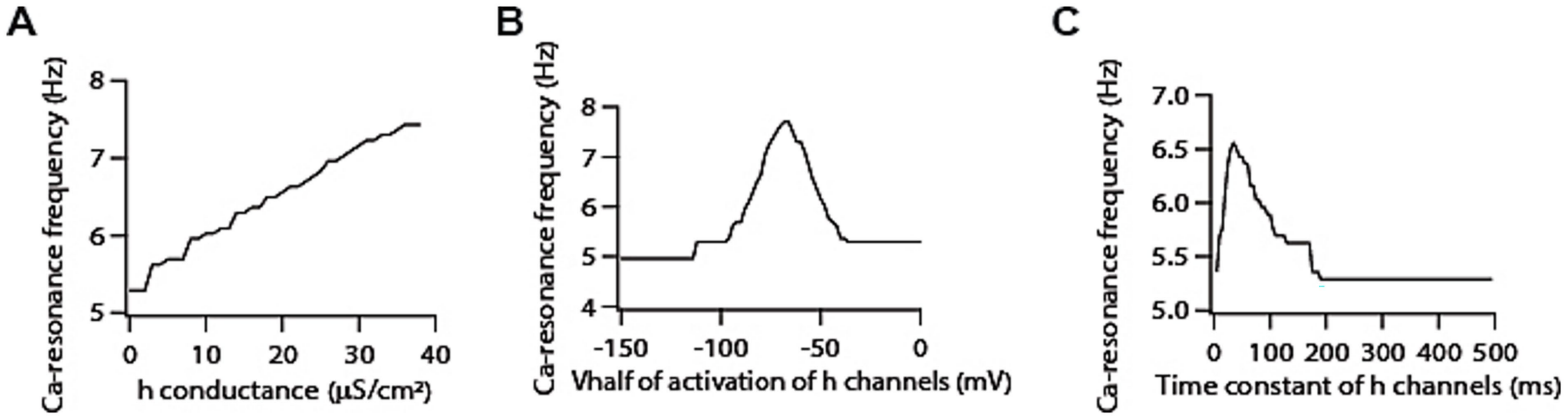
Co-expression of h channels modifies T-type calcium channels-mediated resonance properties. **(A)** Increasing h conductance results in an increase in calcium resonance frequency. **(B)** Shifting *V*_1/2_ of activation of h conductance produces bell shaped like curve for calcium resonance frequency. **(C)** Increasing activation time constant of h conductance results in a decrease in calcium resonance frequency.

First, we co-expressed h channels along with T-type calcium channels and tested their role in determining calcium resonance frequency. We found that increasing h conductance in the presence of T-type calcium conductance led to increase in calcium resonance frequency (Figure 3A). Since it has been shown that *V*_1/2_ of activation of h channels hyperpolarizes along the somato-apical dendritic axis (Magee, 1998), it is important to understand the dependence of calcium resonance frequency on activation curve of h channels. Therefore, we tested the sensitivity of calcium resonance frequency on activation curve of h channels. This showed that shifting the activation curve of h channels along the voltage range produced a bell-shaped like curve for calcium resonance frequency (Figure 3B). On the other hand, increasing the activation time constant of h channels shifted the calcium resonance frequency distribution, causing a leftward skew, and overall decrease in calcium frequency resonance (Figure 3C). These results are expected, as h conductance contributes to voltage resonance by functioning as a high pass filter, and are in line with h channels mediated voltage resonance (Narayanan and Johnston, 2007).

**Figure 4:**
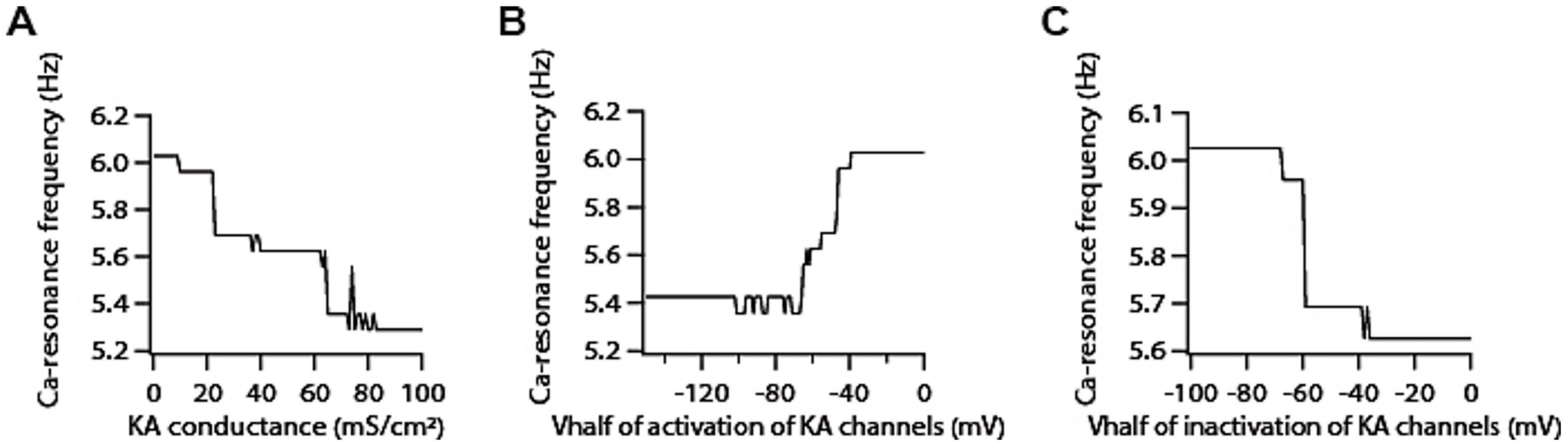
Co-expression of A-type K^+^ channels modifies T-type calcium channels-mediated resonance properties. **(A)** Increasing A-type K^+^ conductance induces a decrease in calcium resonance frequency. **(B)** Shifting *V*_1/2_ of activation of A-type K^+^ conductance in depolarized direction increases calcium resonance frequency. **(C)** Shifting *V*_1/2_ of inactivation of A-type K^+^ conductance in depolarized direction reduces calcium resonance frequency.

Next, we examined the role of A-type K^+^ channels in determining calcium resonance frequency. Given that A-type K^+^ channels act as leak conductance in modulating voltage resonance frequency (Rathour et al., 2016; Rathour and Narayanan, 2012), we suspected that increasing A-type K^+^ conductance would lead to an increase in calcium resonance frequency. To our surprise, we found that increasing A-type K^+^ conductance decreases calcium resonance frequency by only a small magnitude (Figure 4A). A-type K^+^ channels have been shown to exhibit variability in their *V*_1/2_ of activation/inactivation within the homogeneous neuronal population. Moreover, there are differences between proximal and distal dendritic A-type K^+^ channels in terms of *V*_1/2_ activation in CA1 neurons (Hoffman et al., 1997). Therefore, we also examined the sensitivity of calcium resonance frequency on these parameters, and found that shifting the *V*_1/2_ of activation of A-type K^+^ conductance in depolarized direction increases calcium resonance frequency (Figure 4B) whereas shifting the *V*_1/2_ of inactivation of A-type K^+^ conductance in depolarized direction reduces calcium resonance frequency (Figure 4C) but these changes in calcium resonance frequencies were quite small. We did similar analyses with different activation/inactivation time constants of A-type K^+^ channels and found that changing their values did not affect calcium resonance frequency (Supplementary Figure S2). Therefore, these results suggest that calcium resonance frequency is largely independent of A-type K + channels.

### Calcium resonance frequency along the somato-apical dendritic axis

So far, we focused on single compartment model where model neuron expressed either T-type calcium channels alone or in conjunction with A-type K^+^ channels or h channels. This setup enabled us to explore the dependence of calcium resonance frequency on various active and passive parameters related to aforementioned voltage gated ion channels and membrane properties. Next, we aimed to explore calcium resonance frequency in a morphologically realistic model of neuron, and examine the calcium resonance frequency map along the somato-apical dendritic trunk of the neuron.

For this, we utilised a previously developed model neuron which expressed gradients of various voltage gated ion channels along the topography of the neuron and was heavily constrained by the experimental statistics related to six intra-neuronal functional maps (Rathour and Kaphzan, 2023; Rathour and Kaphzan, 2022; Rathour and Kaphzan, 2024; Rathour and Narayanan, 2014). First, we recorded local voltage and calcium response after delivering the chirp stimulus at three different locations (colored dots in Figure 1A) along the somato-apical dendritic trunk. As expected, the shape of the voltage resonance frequency distributions at soma and around 150 μm was similar in the sense that both have peaks in the theta range (~5 Hz) and comparable bandwidths, although absolute amplitudes were different, as the gain of the 150 μm curve was lower (Figures 1B,D). Whereas at ~300 μm from the soma, voltage resonance frequency increased heavily exhibiting a pronounced higher pass segment, possibly due to the higher local h conductance and lower membrane input resistance, yielding a positive slope over the low–mid frequencies before the eventual high frequency roll off (Figures 1B,D), as shown experimentally (Narayanan and Johnston, 2007; Rathour et al., 2016). When we looked at the calcium resonance frequency at the aforementioned three locations, we found that calcium resonance frequency followed the trend of voltage resonance frequency with regard to location effects. Specifically, calcium resonance frequencies were similar at the soma and around 150 μm (Figures 1C,E). Similarly, calcium resonance frequency increased at around 300 μm from the soma (Figures 1C,E), although this increase was smaller compared to increase in voltage resonance frequency. Intrigued by these results, we investigated how does the entire calcium resonance frequency map along the somato-apical dendritic trunk look like, and how does it compare with voltage resonance frequency map. For this, we computed resonance frequencies related to calcium and voltage dynamics along the entire somato-apical dendritic trunk. This simulation showed that calcium resonance frequency remains similar up to 200 μm from the soma and increases beyond that point (Figure 1F). In addition, similar to our previous observation, this increase in calcium resonance frequency was small compared to the increase in voltage resonance frequency (Figure 1F).

## Discussion

In this study, using the modeling framework, we explored the intrinsic calcium response dynamics of hippocampal neurons during oscillatory input. In doing so, we found that along with voltage resonance, T-type calcium conductance was able to sustain calcium resonance on its own. However, unlike voltage resonance, we showed that calcium resonance frequency was largely independent of T-type calcium conductance magnitude (Figure 2C). Furthermore, we showed that co-expression of either A-type K^+^ or h channels with T-type calcium channels modulated calcium resonance frequency (Figures 3, 4). Moreover, intrinsic calcium response dynamics did not simply follow voltage dynamics; rather, there was a dissociation between the two, suggesting that calcium signaling operates through mechanisms beyond membrane potential fluctuations and highlighting a distinct regulatory mechanism. In the following sections, we discuss the implications of these findings.

One of the important results was the finding that unlike voltage resonance frequency, calcium resonance frequency showed only a small increase when T-type calcium conductance was increased by several fold (Figure 2C), and calcium resonance frequency remained within the 5–7 Hz range. This frequency range is the most commonly observed frequency range during theta oscillations *in vivo* (Buzsaki, 2002; Buzsaki, 2006). It could be that calcium entry from T-type calcium channels is required in this frequency range for normal functioning/trafficking of other ion channels. Such coupling between T-type calcium and A-type K^+^ channels has been shown where calcium entry from T-type calcium channels affect A-type K channels properties through their interaction with A-type K^+^ channels auxiliary subunit KChIPs (Anderson et al., 2010). Hence, keeping the calcium resonance frequency largely independent of T-type calcium conductance magnitude would provide a unique way for normal functioning of coupled ion channels. Furthermore, the finding that oscillatory calcium dynamics is dissociated from membrane potential fluctuations and does not simply follow voltage signal suggests an evolution of a failsafe mechanism against some pathological insults, where changes in certain types of VGICs properties could affect voltage dynamics but still keeping T-type calcium channels mediated calcium response dynamics more stable, maintaining homeostasis.

Homeostasis of neuronal intrinsic properties is extremely important for normal brain functioning (Marder and Goaillard, 2006; Marder, 2011; Rathour and Narayanan, 2019). Alteration of these properties could lead to pathological conditions that impair cognitive performance. A canonical way of achieving homeostasis of intrinsic properties has been seen through the lens of adjustment to the single type of VGIC properties, which mediate particular intrinsic properties. However, research into past couple of decades have clearly shown that other VGICs, which cannot mediate these particular intrinsic properties on their own but could modulate these intrinsic properties, could provide additional solutions for achieving homeostasis of intrinsic properties by providing non-canonical ways (Marder and Goaillard, 2006; Marder, 2011; Rathour and Narayanan, 2019). To this end, our results show that although calcium resonance is mediated by T-type calcium channels, it is also modulated by the presence of A-type K^+^ and h channels along with their associated parameters and passive membrane properties (Figures 3, 4). Therefore, it stands to reason that presence of A-type K^+^ and h channels along with passive membrane parameters could provide additional non-canonical ways of achieving homeostasis of calcium resonance.

Although calcium resonance defines that at a particular input frequency calcium response is maximal, but the maximal calcium response in our study was found to be small, usually in tens of nanomolar range, which might not induce any physiologically relevant changes in neuronal properties. Several reasons could explain this small deflection in intra-cellular calcium concentration. One reason could be that the input signal used in this study was of small amplitude producing up to 5 mV bidirectional (depolarizing or hyperpolarizing) voltage deflections from the resting potential, while *in vivo* condition the membrane potential oscillations are of larger amplitudes that could initiate action potential firing (Harvey et al., 2009). This is particularly more pronounced in dendrites where they can initiate dendritic spike (London and Hausser, 2005; Buzsaki, 2002). Therefore, large amplitude membrane potential oscillations coupled with action potential firing and/or dendritic spiking will induce large amplitude changes in intra-cellular calcium levels that could be physiologically relevant for altering neuronal properties. Other reason for the small deflection in intra-cellular calcium level could be the time scale of the input signal used. In our study, input signal spanned 0–15 Hz of frequency in 15 s, while on the behavioral time scale a small band of frequencies could last for the several seconds to minute. Under this scenario, accumulation of intra-cellular calcium will be much larger compared to calcium levels in response to our single input chirp signal.

Although the herein study is entirely computational, the aforementioned predictions could be examined in experimental settings. Calcium resonance can be tested in acute hippocampal slice preparations by combining whole-cell recordings with two-photon calcium imaging of high-performance genetically encoded indicators. A constant-amplitude sinusoidal chirp can be applied via a whole-cell patch-clamp while imaging *ΔF/F* at the soma and in distinct regions of interest along the apical dendrite to allow the construction of calcium impedance profile in each region. Such dendritic measurements at ~50–300 μm can test the map of calcium resonance as predicted by the model. Additionally, selective pharmacological or optogenetic modulation of T-type calcium channels, A-type K^+^ channels, or h-channels would allow for direct testing of the described channel-specific contributions. Together, these experimental strategies can provide feasible avenues to validate the abovementioned computational predictions and to establish calcium resonance as a measurable physiological phenomenon.

Beyond a deeper comprehension of endogenous neuronal physiology and its effects on neural circuits and brain functioning, our study might also contribute to a better understanding of brain stimulation therapies that are based on oscillatory stimulations such as transcranial alternating current stimulation (tACS). tACS is a non-invasive brain stimulation technique where electrodes are placed on the scalp and alternating current is applied. This causes membrane potential of the neurons to oscillate, especially in the distal regions of dendrites and axons, thus comprehensively simulating the in vivo like conditions. tACS has been widely used to treat various brain-related disorders like depression, chronic pain, Parkinson’s disease and others. tACS is usually applied for 20–30 min which can cause a gradual increase in intra-cellular calcium levels, subsequently altering neuronal physiology causing clinically beneficial effects. One of the important parameters of tACS that needs to be chosen when applied in a clinical setting is the stimulation frequency, and it is estimated that only a certain band of stimulation frequency has beneficial effects. Our results provide an explanation for the importance of choosing a distinct stimulation frequency within the theta frequency band. As we have shown, the calcium entry through T-type calcium channels is dependent upon the injected current frequency and only at a particular frequency calcium response is maximum. This suggests that during tACS, stimulating in a frequency that is in the vicinity of calcium resonance frequency (5–7 Hz) would provide an ideal set up for enhancing the accumulation of calcium influx over time through T-type calcium channels, making these frequencies clinically efficient (Debnath et al., 2024; Kaiser et al., 2025).

## Data Availability

The raw data supporting the conclusions of this article will be made available by the authors, without undue reservation.

## Supplementary material

SUPPLEMENTARY FIGURE S1 Dependence of T-type calcium channels mediated resonance on passive parameters. **(A)** Single compartmental model used in this study. **(B)** Increasing calcium decay time constant reduces T-type calcium channels mediated resonance frequency. **(C)** Increasing membrane resistance produces small increase in T-type calcium channels mediated resonance frequency. **(D)** Increasing membrane capacitance decrease T-type calcium channels mediated resonance frequency.

SUPPLEMENTARY FIGURE S2 Changes in A-type K^+^ channels kinetics do not alter calcium resonance frequency. **(A)** Increasing A-type K^+^ activation time constant does not affect calcium resonance conductance induces a decrease in calcium resonance frequency. **(B)** Increasing A-type K^+^ inactivation time constant does not affect calcium resonance frequency.
