# Supplementary figures and images for: Intrinsic calcium resonance and its modulation: insights from computational modeling
Source: Front Comput Neurosci. 2025 Sep 18;19:1669841. doi: 10.3389/fncom.2025.1669841 (PMC12488614; doi:10.3389/fncom.2025.1669841)

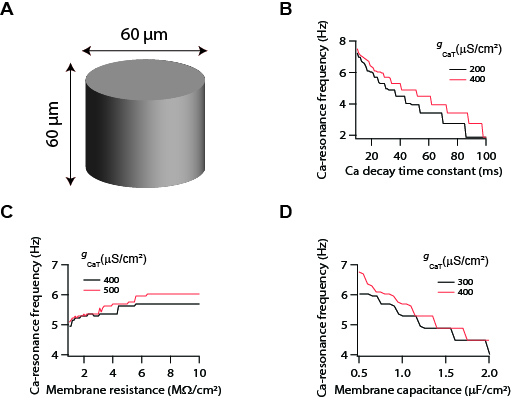

Supplement: SUPPLEMENTARY FIGURE S1 — Dependence of T-type calcium channels mediated resonance on passive parameters. (A) Single compartmental model used in this study. (B) Increasing calcium decay time constant reduces T-type calcium channels mediated resonance frequency. (C) Increasing membrane resistance produces small increase in T-type calcium channels mediated resonance frequency. (D) Increasing membrane capacitance decrease T-type calcium channels mediated resonance frequency. [file Image_1.JPEG]

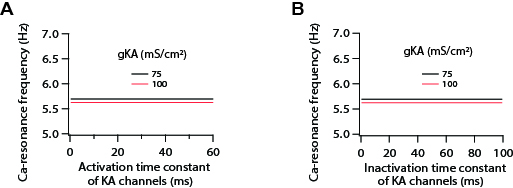

Supplement: SUPPLEMENTARY FIGURE S2 — Changes in A-type K+ channels kinetics do not alter calcium resonance frequency. (A) Increasing A-type K+ activation time constant does not affect calcium resonance conductance induces a decrease in calcium resonance frequency. (B) Increasing A-type K+ inactivation time constant does not affect calcium resonance frequency. [file Image_2.JPEG]
